# Intraspecific functional trait variation and performance of *Populous tremuloides*

**DOI:** 10.3389/fpls.2013.00527

**Published:** 2013-12-24

**Authors:** Florian Fort

**Affiliations:** Institut National de la Recherche Agronomique, UMR 1248 AGIRCastanet-Tolosan, France

**Keywords:** fine root morphology, genetic variation, intraspecific variation, relative growth rate, specific leaf area, root tissue density, root functional trait

The fact that plant-trait and functional-trait syndromes can describe plant growth strategies, and as a result their ecological requirements, has gained considerable importance in ecology (Grime et al., 1997; Westoby et al., 2002). At the global scale, the existence of a major axis of specialization among plants has been determined by measuring leaf functional traits; this axis highlights a fundamental trade-off between rapid acquisition of resources and conservation of resources within well-protected tissues (Díaz et al., 2004; Wright et al., 2004). Díaz et al. (2004) demonstrated that water and nutrient stresses lead to trait-syndrome convergence, with conservative species most present in stressful growth conditions, while acquisitive ones most present in to non-stressful growth conditions. This result highlight the functional traits capacity to explain species adaptation to different growth conditions. Moreover, despite the evidence of a large functional traits differentiation within species and the importance of trait variability within a species to establish itself in different habitats (Violle et al., 2012), functional strategies generally have been identified at the species level and did not incorporate within species traits variability.

The fact that functional traits measurement allowed the definition of plant strategies, and as a result their ability to develop biomass in a variety of growth conditions, led Garnier and Navas (2011) to recommend this approach for agricultural and forestry systems to maximize services of these types of production, and to reduce their environmental drawbacks. However, several questions remain: (i) Do the species used in forestry and agro-systems display different trait syndromes among genotypes?; (ii) Is trait-value variation among genotypes related to genetic differences? and (iii) Are the strategies related to performance of the genotype?

To answer these questions Hajek et al. (2013) test if leaf as well as root functional traits are related to *Populus* varieties performance. In order to do that, they grew seven demes, i.e. an assemblage of closely related individuals (Gilmor and Gregor, 1939), of two conspecific subspecies, the European *Populus tremula* and one deme of the North American *P. tremuloides* in monoculture stands in a common garden in the Solling Mountains (Germany). For each deme, roots were collected in the upper 30 cm of the mineral soil at a stem distance of 15–30 cm from 18 to 20 randomly chosen juvenile trees. Root functional traits, including specific root length (SRL, m.g^−1^), specific root area (SRA, cm^2^ g^−1^), root tissue density (RTD, g cm^−3^), root tip abundance, root diameter, and *N* content were measured on fine roots (<2 mm) according to Cornelissen et al. (2003). Simultaneously with root sampling, leaves were collected from the same trees to measure leaf area (cm^2^), specific leaf area (SLA, cm^2^*g*^−1^), and N and C contents.

Hajek et al. (2013) showed strong intraspecific variation in nearly all functional traits; only RTD did not vary among demes. These results highlight that although root traits can describe species' strategies well, they may also allow populations within a species to be differentiated. This interpretation is strengthened by the fact that despite the small number of demes considered, two roots traits (root tip abundance and RTD) and two leafs traits (leaf size and SLA) appeared to be linked to genetic variation among demes. Moreover, some demes showed particularly high within-deme trait variation, suggesting the existence of strong within-deme genetic diversity. This result challenge the idea that a species should be described by one trait value and opens interesting perspectives in plant breeding by highlighting the existence of strong within-species trait diversity linked to genetic differentiation, which is a prerequisite for breeding based on functional traits. The authors did not highlight links between above-ground relative growth rates (RGR) and root traits, while leaf traits appeared to be linked to the RGR but are poor predictors of it. They hypothesized that this was due to a stronger relation between root traits and below-ground RGR than above-ground RGR, showing the need for more whole-plant holistic studies to better understand plant functioning. These links were tested in a single growth environment. Root functional traits should be linked more to species performance under conditions where water and nutrient availability limits plant growth and thus where different root strategies should result in contrasting performance (Zhu et al., 2010).

In summary, study by Hajek et al. (2013) demonstrated the existence of significant intraspecific trait variation linked to genetic differences among demes. They also demonstrated that our knowledge about the links between traits and plant performance must be improve. It is hoped that the Hajek et al. (2013) study will stimulate more research efforts to clarify the relations among trait values, plant relatedness, plant performance and environmental factors, in order to let the concept of functional traits be functional in forestry and agronomy.
